# Reducing the Rate of Treatment Disruptions Through a Digital Structured Exercise and Mind–Body Program During Systemic Cancer Therapy: A Secondary Analysis of a Randomized Clinical Trial

**DOI:** 10.3390/cancers18060984

**Published:** 2026-03-18

**Authors:** Karolina L. Bryl, Marco Santos Teles, Raymond E. Baser, Jun J. Mao, Bobby Daly

**Affiliations:** 1 Integrative Medicine and Wellness Service, Department of Medicine, Memorial Sloan Kettering Cancer Center, New York, NY 10065, USA; 2 Department of Epidemiology and Biostatistics, Memorial Sloan Kettering Cancer Center, New York, NY 10065, USA; 3 Thoracic Oncology Service, Department of Medicine, Memorial Sloan Kettering Cancer Center, New York, NY 10065, USA

**Keywords:** integrative medicine, treatment discontinuation, treatment disruptions, cancer, exercise, mind–body therapies, digital

## Abstract

Background/Objectives: Treatment disruptions and discontinuations during systemic cancer therapy are common and can compromise treatment delivery and outcomes. Structured exercise and mind–body interventions improve cancer-related symptoms, but their impact on treatment disruptions and discontinuations remains unclear. This secondary analysis of the IMPROVE trial evaluated whether participation in Integrative Medicine at Home (IM@Home), a digital multimodal mind–body and structured exercise program, was associated with differences in treatment discontinuation and related treatment disruption outcomes among patients undergoing systemic therapy. Methods: A total of 127 adults with solid tumors were randomized to IM@Home (n = 64) or enhanced usual care (EUC; n = 63) for 12 weeks. Treatment discontinuation, dose delays, dose reductions, and overall treatment disruptions were compared between arms using chi-square tests and regression models adjusted for cancer type and disease stage. Results: In unadjusted analyses, treatment discontinuation occurred less frequently in the IM@Home group compared with EUC (9.4% vs. 22.6%; *p* = 0.043), but this association was attenuated after adjustment for cancer type and disease stage (aOR 0.41, 95% CI 0.13–1.17; *p* = 0.105). The proportion of patients experiencing any treatment disruption, as well as rates of dose delays and dose reductions, did not differ significantly between groups (*p* = 0.16, *p* = 0.18, and *p* = 0.85, respectively). In contrast, IM@Home participants experienced fewer treatment disruption events per patient (adjusted RR 0.58, 95% CI 0.35–0.96; *p* = 0.036). Conclusions: These exploratory findings suggest that digital structured exercise and mind–body programs may help mitigate treatment interruptions during systemic cancer therapy and should be explored further in an adequately powered prospective trial to confirm these promising findings.

## Simple Summary

Cancer treatments can be delayed, reduced, or stopped due to side effects like fatigue, which can affect a patient’s treatment outcomes. This study explored whether a digital exercise and mind–body program, Integrative Medicine at Home (IM@Home), could help reduce treatment discontinuations and treatment disruptions. A total of 127 patients with cancer reporting moderate to severe fatigue were randomly assigned to either a 12-week IM@Home program or enhanced usual care (EUC) group. We examined whether participation in IM@Home affected treatment discontinuation and other treatment disruptions, including dose delays and reductions. Our findings suggest that patients who participated in IM@Home had fewer repeated treatment interruptions during the study period. Although the program did not clearly reduce the chance of stopping treatment altogether, it appeared to help reduce ongoing disruptions once challenges occurred. These early findings suggest that supportive digital programs may help patients better manage symptoms and stay on track with treatment. More research is needed to confirm these results.

## 1. Introduction

Patients undergoing systemic therapy for cancer frequently experience treatment disruptions, including treatment discontinuations, dose delays, and dose reductions, which can compromise treatment delivery and long-term outcomes [1,2]. Treatment disruptions arise from multiple factors, including treatment-related toxicities, patient-related factors (e.g., general health, personal reasons, financial toxicity), and issues related to disease progression [1]. Prior studies suggest that uncontrolled symptoms—such as fatigue, pain, and psychological distress, which are highly prevalent during systemic therapy—often lead to treatment disruptions [3,4,5,6,7]. Close monitoring of symptoms and proactive management of side effects are considered important components of supportive care aimed at maintaining treatment delivery and improving patient outcomes [8,9,10,11].

Exercise and mind–body therapies, such as yoga, mindfulness, and Tai Chi, have been shown to effectively alleviate cancer- and treatment-related symptoms across the cancer continuum [12,13,14,15,16,17]. Meta-analyses consistently demonstrate the benefits of exercise in reducing fatigue, enhancing physical function, and promoting disease-free survival [18,19,20,21]. Similarly, mind–body therapies, such as yoga or meditation, improve health-related quality of life and reduce fatigue, insomnia, anxiety, and depression [15,22,23,24]. These therapies are endorsed by major oncology organizations, including the American Society of Clinical Oncology and the National Comprehensive Cancer Network, for symptom management in cancer care [24,25,26]. However, despite their established benefits for cancer-related symptoms and quality of life [24,25,27,28], it remains unclear whether improvements in symptom burden resulting from these interventions translate into improved treatment delivery, such as fewer discontinuations, dose delays, or dose reductions [29,30,31].

To address this gap, we conducted a secondary analysis of the Integrative Medicine for Patient-Reported Outcomes, Values, and Experience (IMPROVE) trial, which examined the impact of Integrative Medicine at Home (IM@Home), a digital mind–body and structured exercise program, on fatigue and comorbid symptoms among patients with solid tumors undergoing systemic therapy [17]. The trial demonstrated that IM@Home significantly reduced fatigue severity, symptom distress, anxiety, and depression compared with enhanced usual care [17], and was associated with reduced acute care utilization [32]. The present study aims to explore whether participation in IM@Home is associated with differences in treatment discontinuations and treatment disruptions, including dose delays and dose reductions, during systemic therapy.

## 2. Materials and Methods

### 2.1. Design

This secondary analysis used data from the IMPROVE trial—a single-center, pragmatic, two-arm, parallel-group basket-design randomized clinical trial (RCT) evaluating IM@Home versus enhanced usual care (EUC) among patients with cancer on systemic therapy who reported moderate or greater fatigue [17]. The study was approved by the Memorial Sloan Kettering (MSK) Institutional Review Board (IRB# 21-369) and registered at clinicaltrials.gov (NCT05053230). All participants provided written informed consent.

### 2.2. Eligibility Criteria

**Figure 1:**
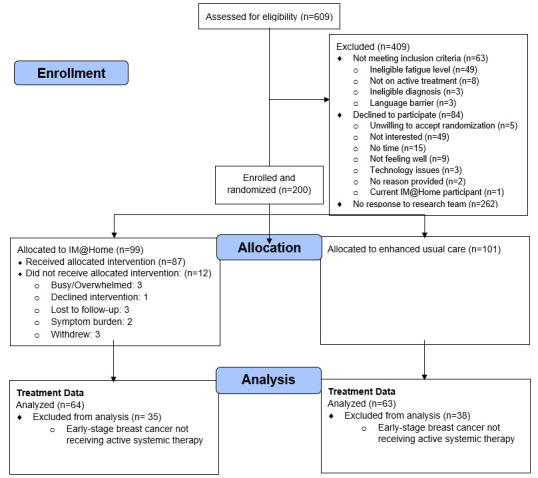
CONSORT Diagram of Participant Flow in the IMPROVE Study. This figure presents a consort diagram detailing the participant flow through the IMPROVE study.

Eligible participants were English-speaking adults (≥18 years) diagnosed with breast, thoracic, gynecologic, or head and neck cancers, or melanoma, undergoing systemic therapy (chemotherapy, immunotherapy, or targeted therapy), reporting a fatigue score ≥ 4 on the “worst fatigue” item of the Brief Fatigue Inventory (0–10 scale, with 10 representing the most severe fatigue [33,34]), with a Karnofsky Performance Status ≥ 60 and life expectancy ≥ 6 months. For this secondary analysis, patients with breast cancer treated with radiation alone were excluded (Figure 1) as the objective was to evaluate patterns in systemic therapy use.

### 2.3. Interventions

Patients were randomized 1:1 to either IM@Home or EUC, stratified by tumor type, for 12 weeks. IM@Home participants received access to 23 live, synchronous mind–body and structured exercise classes delivered via Zoom. EUC participants received standard care and access to 17 pre-recorded, on-demand audio or video recordings for meditation, guided imagery, and relaxation. Detailed intervention description has been published elsewhere [17].

### 2.4. Outcome Measures

The primary outcome was treatment discontinuation, defined as cessation of systemic therapy, including discontinuations due to progression of disease (POD), adverse events, or death, relative to the start of the intended systemic regimen, consistent with prior studies [35].

Secondary outcomes included *dose delays*, *dose reductions*, and *treatment disruptions*. *Dose delays* were defined as a delay of 7 or more days in the administration of any chemotherapy, immunotherapy, targeted therapy cycle relative to the standard day of administration, consistent with prior oncology studies [35,36,37,38,39]. *Dose reductions* were defined as any dose reduction of any chemotherapy, immunotherapy, or targeted therapy agent relative to the standard dose in any treatment cycle [35,36,37,38,39]. Missing doses were recorded when at least one antineoplastic agent from the planned regimen was not administered in a cycle. Consistent with previous studies, any missed dose was classified as a dose delay [35,36,37,38,39]. The “standard day” of administration and “standard dose” for each systemic agent were determined based on the treating oncologist’s prescribed regimen documented in the electronic health record (EHR), including cycle length and planned dosing schedule at treatment initiation. When applicable, standard schedules were cross-referenced with institutional treatment protocols and published guidelines to ensure consistency. Deviations were defined relative to the intended regimen for each individual patient rather than a uniform protocol across cancer types. *Treatment disruptions* were defined as any unplanned dose delays, dose reductions, or treatment discontinuations, relative to the start of the intended systemic regimen, consistent with prior studies [1,2,3]. Treatment disruptions were recorded as both binary variables (any disruption: yes/no) and count variables (number of disruption events per patient). For each patient, the systemic agent involved, as well as the timing and reason for each disruption, was extracted from the EHR and adjudicated by two reviewers (MST, BD) blinded to participants’ treatment assignment.

### 2.5. Statistical Analysis

Descriptive statistics were used to summarize participant characteristics and primary and secondary outcomes. Categorical variables were summarized as frequencies and percentages, and continuous variables as means with standard deviations or medians with interquartile ranges, as appropriate.

Proportions of patients experiencing treatment discontinuations or disruptions were compared between IM@Home and enhanced usual care (EUC) using Pearson’s Chi-squared tests.

Logistic regression models were additionally used to estimate odds ratios (ORs) and 95% confidence intervals (CIs) for treatment discontinuation, dose delays, dose reductions, and any treatment disruption, adjusting for cancer type and disease stage. These adjusted analyses were conducted to account for potential imbalance in disease stage between study arms.

To evaluate the frequency of treatment disruption events, negative binomial regression models were used to compare the rate of treatment disruptions per patient between arms. Although our disruption event counts showed evidence of only mild overdispersion, we conservatively utilized negative binomial regression instead of Poisson regression because the former is more appropriate for overdispersed count data. Both unadjusted and adjusted models were estimated. Adjusted models included cancer type and disease stage. Rate ratios (RRs) and 95% CIs were reported.

Separate negative binomial models were used to evaluate rates of dose delays and dose reductions per patient. As treatment discontinuation can occur at most once per patient, it was not analyzed in a separate negative binomial model. Adjusted models for dose reductions could not be reliably estimated due to sparse events and model non-convergence.

All statistical tests were two-sided with a significance threshold of α = 0.05. Because this was a secondary analysis of a trial not originally powered for treatment disruption outcomes (discontinuation, dose reductions, and delays), these analyses are exploratory in nature. Given the modest sample size and relatively low frequency of some events, particularly treatment discontinuation and dose reductions, emphasis was placed on effect size estimates and corresponding 95% CIs to inform precision, rather than reliance solely on statistical significance testing. Analyses were conducted using R version 4.5.0.

## 3. Results

### 3.1. Participants

Of 127 patients included in this analysis (64 [50.4%] IM@Home; 63 [49.6%] EUC), mean age was 64 (SD = 12) years, 108 (85.0%) were female, 105 (82.7%) were white, and 5 (3.9%) were Hispanic or Latino. Cancer types included: 49 (38.6%) thoracic, 43 (33.9%) gynecologic, 25 (19.7%) head and neck, and 10 (7.9%) melanoma. Fifty-five (43.3%) had early-stage or locally advanced disease and 72 (56.7%) had metastatic disease. Treatments included: 60 (47.2%) chemotherapy, 17 (13.4%) immunotherapy, and 53 (41.7%) targeted therapy (Table 1). A higher proportion of participants in the EUC arm had stage IV disease compared with IM@Home (63.5% vs. 50.0%); baseline disease stage was therefore included as a covariate in adjusted models. Patients could be on more than one category of systemic therapy concurrently.

### 3.2. Number and Type of Treatment Discontinuations and Disruptions

IM@Home participants experienced significantly fewer treatment discontinuations compared to EUC (9.4% vs. 22.6%, *p* = 0.043). Among the 127 participants, 42.1% experienced at least one treatment disruption during the 12-week study period (Table 2). The proportion of patients with any treatment disruption was similar between arms (35.9% for IM@Home vs. 48.4% for EUC; *p* = 0.16). Treatment discontinuations were more frequent in the EUC arm, mainly due to disease progression. Dose delays were most often caused by infections and other toxicities, with higher frequencies observed in the EUC arm. Dose reductions had similar distribution across arms (Table 3).

### 3.3. Effect of IM@Home on Treatment Discontinuations and Disruptions

In logistic regression models (Table 4) adjusted for cancer type and disease stage, treatment discontinuation odds were lower in the IM@Home group, but this did not reach statistical significance (adjusted odds ratio [aOR] 0.41, 95% CI 0.13–1.17; *p* = 0.105). No statistically significant difference was observed in the odds of experiencing dose delays (aOR 0.54, 95% CI 0.22–1.25; *p* = 0.154), dose reductions (aOR 0.96, 95% CI 0.33–2.86; *p* = 0.944), or any treatment disruption (aOR 0.62, 95% CI 0.30–1.29; *p* = 0.203) between arms.

### 3.4. Rates of Treatment Disruption, Dose Delay, and Dose Reduction Events

Treatment discontinuation was examined separately as a binary outcome and is reported descriptively in Table 2 and in adjusted logistic regression models in Table 4, given that discontinuation can occur at most once per patient. The analyses and results below therefore focus on rates of recurrent treatment disruption events per patient.

The IM@Home group had a significantly lower rate of treatment disruptions per patient compared to EUC (rate ratio [RR] 0.58, 95% CI 0.35–0.96; *p* = 0.035; Table 5). This finding remained consistent after adjustment for cancer type and disease stage (RR 0.58, 95% CI 0.35–0.96; *p* = 0.036). For dose delays, unadjusted and adjusted models suggested lower rates among IM@Home participants, but these differences did not reach statistical significance (adjusted RR 0.55, 95% CI 0.27–1.09; *p* = 0.091). Dose reductions showed no significant differences between arms, and adjusted models could not be reliably estimated due to sparse events.

## 4. Discussion

Systemic cancer therapy is frequently accompanied by substantial symptom burden that can contribute to treatment discontinuations and disruptions and compromise outcomes [10,40]. In this secondary analysis of the IMPROVE trial, participation in IM@Home, a digital, multimodal structured exercise and mind–body exercise program, was associated with a lower rate of treatment disruption events per patient compared with EUC. Although unadjusted analyses suggested fewer patients experienced any treatment discontinuation in the IM@Home arm, this association was attenuated after adjustment for cancer type and disease stage and was not statistically significant, likely reflecting limited power due to the small number of events. Although a similar proportion of patients in both arms experienced at least one treatment disruption, patients assigned to IM@Home had fewer disruptions per patient, resulting in improved treatment continuity. While adjusted odds ratios for experiencing any disruption were not statistically significant, effect estimates consistently favored IM@Home across endpoints, supporting a meaningful signal that structured, symptom-focused integrative medicine support may help stabilize patients on systemic therapy and reduce cascading interruptions in care.

A key distinction in this analysis is between the likelihood of experiencing any treatment disruption and the frequency of disruption events per patient. Adjusted logistic regression models did not demonstrate a statistically significant difference in the odds of experiencing at least one disruption. In contrast, negative binomial models revealed a significantly lower rate of disruption events per patient among IM@Home participants. This pattern suggests that the intervention may have a relatively limited effect on preventing the first disruption, which is often driven by disease progression or unavoidable toxicity, but may reduce subsequent or cascading interruptions once symptoms emerge [41,42]. Clinically, this distinction is meaningful. Reducing recurrent disruptions may reflect improved symptoms, better self-management, preserved functional reserve, and/or enhanced engagement with care, all of which could help stabilize patients during ongoing systemic therapy [3,43].

The observed reduction in treatment discontinuations in unadjusted analyses is noteworthy, as early cessation can preclude optimal therapeutic benefit [44]. However, discontinuation occurred relatively infrequently, limiting precision and widening confidence intervals, and this study was not powered to detect differences in such events. After adjustment for disease stage—which is closely linked to disease progression-driven discontinuation—the association was attenuated, suggesting that baseline stage partially accounted for between-arm differences. Notably, the enhanced usual care group had a greater proportion of patients with stage IV disease, and the majority of discontinuations in this arm were attributed to disease progression. It is therefore plausible that worse symptom control in the enhanced usual care arm may have prompted earlier imaging to evaluate for progression, leading to earlier detection of disease advancement and subsequent treatment discontinuation. This interpretation aligns with clinical practice guidelines for monitoring metastatic disease, which recommend reassessment of disease activity in patients with new or worsening signs or symptoms regardless of the interval since prior imaging [45]. The absence of a clear effect on dose reductions, which are often driven by laboratory abnormalities or worsening symptoms, supports a cautious interpretation: integrative medicine programs may influence treatment adherence rather than prevent all forms of treatment disruptions. These findings underscore that discontinuation outcomes may be heavily influenced by disease biology, with the timing of progression detection potentially modulated by symptom burden and clinical decision-making around imaging, and with supportive interventions playing a more limited role alone [44,46].

This secondary analysis suggests that participation in a digital exercise and mind–body program may reduce treatment disruptions, particularly repeated disruptions, among patients receiving systemic cancer therapy. Prior trials and meta-analyses of exercise and mind–body therapies in oncology have primarily focused on symptom management, functional outcomes, and quality of life, with limited evaluation of downstream clinical outcomes such as treatment adherence or continuity [16,47,48,49,50]. One retrospective study found that patients with advanced cancer who participated in supervised exercise during treatment experienced fewer treatment disruptions compared to historical controls [2]. Our findings extend this literature by leveraging a randomized design and demonstrating that a fully virtual, multimodal program may be associated with improved treatment continuity.

Although this secondary analysis was not designed to test mechanisms, the observed pattern of results is consistent with prior evidence linking symptom burden to treatment tolerability. Treatment disruptions are frequently driven by disease progression [44] or treatment-related toxicities [51], but symptom clusters—fatigue, pain, and psychological distress—are also prevalent during systemic therapy and may limit patients’ ability to tolerate and adhere to planned treatment regimens [3,5,40]. In the parent IMPROVE trial, IM@Home significantly reduced fatigue severity, symptom distress, anxiety, and depression during active treatment [17]. In the current analysis, IM@Home participants experienced approximately 40% fewer treatment disruption events per patient, suggesting that improved symptom control may have helped prevent recurrent or cascading interruptions once symptoms emerged. Exercise may help preserve physical function and mitigate treatment-related deconditioning [52], while mind–body practices may reduce anxiety and depressive symptoms that interfere with treatment engagement [53,54]. Together, these pathways provide a plausible explanation for the observed improvement in treatment continuity, although these mechanisms require formal testing in future studies.

Beyond direct symptom effects, participation in exercise and mind–body interventions may enhance self-efficacy in managing side effects. Engagement in a structured program may increase patients’ confidence in coping with fatigue, pain, and emotional distress, potentially reducing treatment interruptions. Improved self-efficacy may also promote earlier symptom recognition and more proactive symptom management behaviors [55,56,57]. Regular class participation may increase attentiveness to bodily signals and encourage timely reporting of emerging symptoms to care teams, enabling supportive management before disruptions escalate into recurrent delays or omissions. The synchronous group format also fosters a sense of connection with others and accountability, providing support, strengthening motivation to remain engaged with treatment, and reinforcing adherence behaviors [57]. Together, these features underscore the potential value of structured symptom-focused digital exercise and mind–body programs to support treatment continuity, while highlighting the need for prospective trials specifically designed to evaluate mechanisms outcomes.

From an implementation perspective, these findings are notable. The digital format of IM@Home reduces logistical and financial barriers and enables participation during periods of fatigue or competing clinical and personal demands, distinguishing it from many digital adherence tools that focus narrowly on treatment reminders or passive symptom monitoring. Furthermore, the capacity to deliver structured exercise and mind–body interventions remotely during active systemic therapy may enhance scalability and integration into routine oncology care. Future work should evaluate implementation outcomes such as reach, adherence, acceptability, and sustainability across diverse settings.

While these findings are promising, they should be interpreted in light of several limitations. First, this was a secondary analysis and was not specifically powered to detect differences in treatment disruptions. Second, the modest sample size and low event rates, particularly for treatment discontinuation and dose reductions, limited statistical power and precision, resulting in wide confidence intervals and attenuation of some associations after adjustment. Accordingly, findings should be interpreted as exploratory and hypothesis-generating, with emphasis on effect size estimates and confidence intervals. Third, treatment disruptions were heterogeneous, and inclusion of progression of disease within discontinuation outcomes may have introduced confounding. Disease progression is unlikely to be influenced by the intervention and may therefore dilute or obscure potential effects on treatment adherence-related disruptions. Although disruptions were rigorously adjudicated from the EHR, reasons for discontinuation were multifactorial and may not fully capture contextual drivers of treatment interruption. Fourth, the study was conducted at a single academic cancer center with a predominantly White, female cohort, which may limit the generalizability of the findings. Fifth, follow-up was limited to 12 weeks, which may be insufficient to capture longer-term treatment continuity patterns or sustained intervention effects across the full course of systemic therapy. Sixth, participants were not blinded to intervention assignment, which may have introduced expectancy effects or influenced symptom reporting and engagement behaviors. Lastly, although baseline disease stage did not differ significantly between study arms, a higher proportion of patients in the EUC group had stage IV disease; disease stage was therefore included as a covariate in adjusted analyses.

Despite these limitations, the study has several strengths, including its randomized design, standardized outcome definitions, rigorous EHR-based adjudication, and comprehensive data capture, which strengthen confidence in the observed associations. Prospective trials powered for treatment delivery endpoints are warranted to confirm these findings and clarify whether symptom improvement mediates effects on treatment continuity.

## 5. Conclusions

In summary, this secondary analysis provides preliminary evidence that a structured digital exercise and mind–body program may reduce treatment disruption events during systemic cancer therapy. While the intervention did not significantly reduce the adjusted odds of experiencing any single disruption, the observed reduction in the disruption rate per patient suggests a potential stabilization effect. Prospective trials powered specifically for treatment delivery and dose-intensity endpoints are warranted to confirm these findings and clarify whether improvements in symptom burden and patient resilience translate into sustained treatment continuity and improved clinical outcomes.

## Figures and Tables

**Table 1:** Participants Characteristics by Study Arm.

Characteristic	Overall (N = 127)	EUC (n = 63)	IM@Home (n = 64)
	N (%)	n (%)	n (%)
**Total**	127 (100)	63 (100)	64 (100)
**Age, mean (SD)**	64.0 (11.6)	64.0 (11.6)	64.0 (11.6)
**Gender**			
Female	108 (85.0)	52 (82.5)	56 (87.5)
Male	19 (15.0)	11 (17.5)	8 (12.5)
**Race**			
White	105 (82.7)	52 (82.5)	53 (82.8)
Black or African American	10 (7.9)	3 (4.8)	7 (10.9)
Asian	7 (5.5)	6 (9.5)	1 (1.6)
Other	5 (3.9)	2 (3.2)	3 (4.7)
**Ethnicity**			
Non-Hispanic	117 (92.1)	60 (95.2)	57 (89.1)
Hispanic or Latino	5 (3.9)	2 (3.2)	3 (4.7)
Not reported	5 (3.9)	1 (1.6)	4 (6.3)
**Education**			
College degree or higher	83 (65.4)	44 (69.8)	39 (60.9)
High school or some college	26 (20.5)	9 (14.3)	17 (26.6)
Unknown	18 (14.2)	10 (15.9)	8 (12.5)
**Cancer type**			
Thoracic	49 (38.6)	24 (38.1)	25 (39.1)
Gynecologic	43 (33.9)	21 (33.3)	22 (34.4)
Head and Neck	25 (19.7)	12 (19.0)	13 (20.3)
Melanoma	10 (7.9)	6 (9.5)	4 (6.3)
**Disease stage at baseline**			
I–III	55 (43.3)	23 (36.5)	32 (50.0)
IV	72 (56.7)	40 (63.5)	32 (50.0)
**Treatment modality** ^1^			
Chemotherapy	60 (47.2)	29 (46.0)	31 (48.4)
Targeted therapy	53 (41.7)	23 (36.5)	30 (46.9)
Immunotherapy	17 (13.4)	10 (15.9)	7 (10.9)

^1^ Patients could receive multiple forms of treatment at time of enrollment. Abbreviations: IM@Home—Integrative Medicine at Home; EUC—enhanced usual care; SD—standard deviation.

**Table 2:** Proportion of Patients Experiencing Treatment Disruptions by Study Arm.

Outcome	Overall (N = 127)	EUC (n = 63)	IM@Home (n = 64)	*p*-Value ^1^
	N (%)	n (%)	n (%)	
**Treatment discontinuation**	**20 (15.9)**	**14 (22.6)**	**6 (9.4)**	**0.043**
Dose delay ^3^	30 (23.8)	18 (29.0)	12 (18.8)	0.18
Dose reduction	17 (13.5)	8 (12.9)	9 (14.1)	0.85
Any treatment disruption	53 (42.1) ^2^	30 (48.4) ^2^	23 (35.9) ^2^	0.16

^1^ Pearson’s Chi-squared test. ^2^ Totals may not equal the sum of subcategories as patients could experience multiple types of disruption events. ^3^ First dose delay only. Abbreviations: IM@Home—Integrative Medicine at Home; EUC—enhanced usual care.

**Table 3:** Reasons for Treatment Disruptions by Study Arm.

Reason for Disruption	Overall (N = 127)	EUC (n = 63)	IM@Home (n = 64)
	N (%)	n (%)	n (%)
**Treatment discontinuation**	**20 (15.7)**	**14 (22.2)**	**6 (9.4)**
Adverse events/toxicities	3 (2.4)	0 (0)	3 (4.7)
Progression of disease	15 (11.8)	12 (19.0)	3 (4.7)
Death	1 (0.8)	1 (1.6)	0 (0)
Other	1 (0.8)	1 (1.6)	0 (0)
**Dose delay**	**30 (23.6)**	**18 (28.6)**	**12 (18.8)**
Adverse events/toxicities	16 (12.6)	11 (17.5)	5 (7.8)
Infections	9 (7.1)	6 (9.5)	3 (4.7)
Other	5 (3.9)	1 (1.6)	4 (6.3)
**Dose reductions**	**17 (13.4)**	**9 (14.3)**	**8 (12.5)**
Adverse events/toxicities	14 (11.0)	6 (9.5)	8 (12.5)
Other	3 (2.4)	3 (4.8)	0 (0)

Abbreviations: IM@Home—Integrative Medicine at Home; EUC—enhanced usual care.

**Table 4:** Adjusted Odds Ratios for Treatment Disruptions (IM@Home vs. EUC).

Outcome	aOR	95% CI	*p*-Value ^1^
**Treatment discontinuation**	**0.41**	**0.13–1.17**	**0.105**
Dose delay	0.54	0.22–1.25	0.154
Dose reduction	0.96	0.33–2.86	0.944
Any treatment disruption	0.62	0.30–1.29	0.203

^1^ Adjusted odds ratios estimated using logistic regression models adjusted for cancer type and disease stage. Abbreviations: IM@Home—Integrative Medicine at Home; EUC—enhanced usual care; aOR—Adjusted odds ratio; CI: confidence interval.

**Table 5:** Rates and Rate Ratios of Dose Delays, Dose Reductions, and Treatment Disruptions per Patient.

Outcome (Events per Patient)	EUC Rate (95% CI)	IM@Home Rate (95% CI)	Rate Ratio (IM@Home vs. EUC) (95% CI)	*p*-Value ^1^
**Unadjusted Negative Binomial Models**				
Treatment disruptions	0.73 (0.53–0.99)	0.42 (0.28–0.63)	0.58 (0.35–0.96)	0.035
Dose delays	0.35 (0.23–0.54)	0.20 (0.12–0.35)	0.57 (0.28–1.12)	0.111
Dose reductions	0.15 (0.08–0.28)	0.13 (0.06–0.25)	0.86 (0.32–2.25)	0.758
**Adjusted Negative Binomial Models** ** ^2^**				
Treatment disruptions	0.72 (0.53–0.99)	0.42 (0.28–0.62)	0.58 (0.35–0.96)	0.036
Dose delays	0.35 (0.23–0.53)	0.19 (0.11–0.33)	0.55 (0.27–1.09)	0.091
Dose reductions ^3^	-	-	-	-

^1^ *p*-values are from the negative binomial model. ^2^ Rates and Rate Ratios (RRs) are adjusted for cancer type and stage. ^3^ Adjusted model for dose reductions did not converge due to sparse events/model non-convergence. Abbreviations: IM@Home—Integrative Medicine at Home; EUC—enhanced usual care; CI: confidence interval.

## Data Availability

The raw data supporting the conclusions of this article will be made available by the authors on request.
